# Fasting Glucose, Haemoglobin A1C (HbA1c), Blood Lipid, and Triglyceride–Glucose Index Parameters in Relation to Subjective Tinnitus

**DOI:** 10.3390/biomedicines13040824

**Published:** 2025-03-30

**Authors:** András Molnár, Viktória Molnár, Panayiota Mavrogeni, Stefani Maihoub

**Affiliations:** 1 Protone Audio Kft., Opera Clinic, Lázár u. 4, H-1065 Budapest, Hungary; 2 Department of Otorhinolaryngology and Head and Neck Surgery, Semmelweis University, Szigony u. 36, H-1083 Budapest, Hungary; 3 Tóth Ilona Health Service, Clinical Medical Institute, Görgey Artúr tér 8, H-1212 Budapest, Hungary; 4 Maihoub ENT Clinic, Aliakmona Street 16, Cy-3117 Limassol, Cyprus

**Keywords:** subjective tinnitus, cardiovascular risk, glucose, HbA1c, total cholesterol, triglycerides, LDL, HDL, triglyceride–glucose index

## Abstract

**Objectives:** This study aimed to analyse blood glucose and lipid levels in subjective tinnitus compared to healthy controls. **Method:** A total of 414 patients with subjective tinnitus and 274 healthy participants were enrolled. All participants underwent comprehensive laboratory testing, which included measurements of fasting blood glucose, HbA1c, and blood lipids. **Results:** The tinnitus group had significantly higher glucose levels than the control group (*p* = 0.024). Additionally, the HbA1c levels were higher in the tinnitus group (*p* = 0.006). Tinnitus patients exhibited elevated levels of total cholesterol (*p* < 0.001), triglycerides (*p* < 0.001), and LDL (*p* < 0.001). However, HDL levels showed no significant difference (*p* = 0.984). Although the triglyceride–glucose index tends to show higher values in the tinnitus group, this difference is statistically insignificant (*p* = 0.688). ROC indicated that LDL had the highest sensitivity for predicting tinnitus (AUC = 0.620; *p* < 0.001, 95% CI: 0.573–0.668). Other parameters showed significant results, excluding the triglyceride–glucose index (AUC = 0.514; *p* = 0.688, 95% CI: 0.443–0.585), which was not a significant predictor. Glucose levels significantly correlate with age (rho = 0.338, *p* < 0.001) and tinnitus onset (rho = 0.107, *p* = 0.034). Additionally, age showed a significant correlation with total cholesterol levels (rho = 0.156, *p* = 0.002) and triglycerides (rho = 0.121, *p* = 0.020) and tinnitus onset (rho = 0.344, *p* < 0.001). Logistic regression analysis revealed that the presence of tinnitus was significantly associated with elevated HbA1c levels (*p* = 0.007) and TG levels (*p* = 0.001). Furthermore, the occurrence of chronic tinnitus was significantly influenced by elevated glucose levels (*p* = 0.026). **Conclusions:** The results showed increased glucose and blood lipid levels in tinnitus. LDL levels exhibited the highest sensitivity in predicting tinnitus. However, the TyG index was not a significant predictor. Given the cross-sectional design of the study, which may limit the results, further longitudinal studies are necessary.

## 1. Introduction

Tinnitus is the perception of sound without an external source, and its prevalence tends to increase with age [1]. Between 5% and 43% of people globally report experiencing tinnitus [2]. In most cases, tinnitus is subjective, meaning that it can only be heard by the individuals experiencing it [3]. Tinnitus can be categorised into primary and secondary types. Primary cases include idiopathic tinnitus and those related to sensorineural hearing loss. When examining the causes of tinnitus, it is common to focus on ear-related issues, such as earwax build-up, acute or chronic inflammations, hearing loss (either sensorineural or conductive), Ménière’s disease, chronic noise exposure, tumours of the vestibulocochlear nerve, and otosclerosis [4]. However, systemic causes of tinnitus are often overlooked. This is particularly essential considering the significant association between tinnitus, cardiovascular events, and all-cause mortality [5]. Therefore, tinnitus can be considered a warning signal from the ears, but it may also indicate potentially life-threatening systemic disorders. Changes in the cardiovascular system significantly impact ear circulation due to a haemodynamic imbalance, making the inner ear particularly susceptible. This highlights the importance of a comprehensive approach to managing tinnitus [6]. Glucose levels are an independent risk factor for cardiovascular mortality, with each 1 mmol/L increase associated with a 17% rise in cardiovascular events [7]. Haemoglobin A1C (HbA1c) is primarily used to diagnose and monitor diabetes mellitus. It provides insights into glucose metabolism over the past 90 days and is also linked to cardiovascular risk [8]. Elevated total cholesterol and low-density lipoprotein (LDL) levels, along with low levels of high-density lipoproteins (HDLs), are well-known factors associated with increased cardiovascular risk [9]. Elevated triglyceride (TG) levels are linked to an increased risk of cardiovascular disease; however, the specific levels at which this risk escalates remain unclear [10]. In order to predict insulin resistance and future cardiovascular risk, the triglyceride–glucose (TyG) index has been formulated. This parameter indicates a link to cardiovascular mortality, myocardial infarction, stroke, and type 2 diabetes mellitus, highlighting the role of insulin resistance in these conditions [11].

Given the strong link between cardiovascular risk and tinnitus, this study aimed to analyse the key predictors of cardiovascular risk. The primary objectives of the study are to determine whether important parameters, including glucose levels, HbA1c, blood lipids, and the TyG index, are elevated in patients with tinnitus. Additionally, the study examines the sensitivity of these parameters in predicting tinnitus.

## 2. Materials and Methods

### 2.1. Study Group

A total of 414 patients suffering from persistent subjective primary tinnitus, both acute and chronic, were enrolled in this study. The basic information about the participants can be found in Table 1. Additionally, 274 participants without tinnitus were included as a control group. The inclusion criteria required that participants have persistent subjective primary tinnitus lasting at least two weeks, with no secondary causes, such as earwax build-up, infections of the outer and middle ears, or otosclerosis, etc. The exclusion criteria included patients under 18 years of age, those who did not provide consent to participate, and individuals with incomplete examination results related to the study. None of the patients or control participants had previously been diagnosed with or treated for diabetes mellitus, insulin resistance, dyslipidaemia, endocrine disorders, or metabolic syndrome, which helped rule out the effects of related medications. All participants provided their informed consent in writing to take part in this study. The investigation adhered to the Declaration of Helsinki and received approval from the Hungarian ETT TUKEB (approval number: BM/29864–1/2024).

### 2.2. Clinical Examinations

All patients with tinnitus underwent a thorough evaluation by an otorhinolaryngologist experienced in tinnitus management. The first step involved gathering a detailed case history along with questions concerning the symptoms of tinnitus. Each patient received a comprehensive otorhinolaryngological examination, which included micro-otoscopy, tympanometry, and acoustic reflex testing, and a physical examination of the nose, throat, larynx, and neck. Following the physical examination, all patients participated in pure-tone audiometry in a soundproof booth, utilising both air- and bone-conduction measurements. Additionally, tinnitus pitch matching was performed for each case. A contrast-enhanced brain MRI was conducted to rule out retrocochlear lesions, such as vestibular schwannoma, and intracranial abnormalities. Carotid and vertebral ultrasonography was performed to detect plaque build-ups or stenosis in the carotid and vertebral arteries. As part of the routine examinations, detailed laboratory testing was also conducted to identify any potential systemic causes of tinnitus as outlined below.

### 2.3. Laboratory Testing

After obtaining consent, patients were instructed to provide blood samples for routine laboratory testing. They were asked to fast before the examination, meaning they should not eat anything beforehand. Compliance with fasting glucose levels was verified using logs maintained by the patients. Venous blood samples were collected in sodium heparin tubes. The samples were sent for comprehensive laboratory testing, which included measuring serum total cholesterol, LDL, HDL, and TG levels, as well as determining HbA1c and glucose levels using the hexokinase method. The laboratory validated the results, and they were carefully reviewed by the examining doctor. The TyG index was calculated using the following formula: Ln (fasting triglycerides [mg/dL] × fasting plasma glucose [mg/dL]/2. Since the parameters were determined in mmol/L during laboratory testing, the values were accurately converted to mg/dL.

### 2.4. Tinnitus Handicap Inventory Questionnaire

The Hungarian Tinnitus Handicap Inventory (THI) questionnaire, which has been previously validated, was used to assess participants’ self-reported severity of tinnitus. The THI consists of 25 items categorised into three scales. The emotional scale includes nine items that address various issues, such as depression, anxiety, and frustration. The functional scale pertains to daily activities, including interpersonal relationships, household management, and stress counselling. The catastrophic category focuses on experiences related to intense feelings of severe illness and loss of control. To determine tinnitus severity, the total score is calculated by summing the points from all three subscales. Based on the scores, tinnitus severity is classified into the following five categories: ‘no handicap’ (0–16 points), ‘mild’ (18–36 points), ‘moderate’ (38–56 points), ‘severe’ (58–76 points), and ‘catastrophic’ (78–100 points) [12,13].

### 2.5. Statistical Analysis

All statistical analyses were conducted using the IBM SPSS version 25 software for Windows (IBM Corporation, Armonk, NY, USA). The normality of the data was assessed using the Shapiro–Wilk test. Continuous variables were reported as means with standard deviations (SD). Statistically significant differences were analysed using the Mann–Whitney *U* test, while categorical data were examined using the Chi-square test. Correlations between parameters were evaluated using Spearman’s correlation test. Additionally, receiver operating characteristic (ROC) curves were generated, and sensitivity values for each parameter were determined based on the area under the curve (AUC). Furthermore, a multinomial logistic regression model was applied as well. The significance level was set at *p* < 0.05.

## 3. Results

The basic demographic data of the study population are summarised in Table 1.

Table 1 reports that there were no statistically significant differences in age (*p* = 0.06) or sex (*p* = 0.81) between the tinnitus and control groups. Therefore, these two groups are suitable for further analysis. A slight predominance of females was observed in both groups. Most cases of tinnitus are characterised by chronic symptoms, with an onset range varying from 0.5 to 360 months. Bilateral tinnitus was observed most frequently, followed by symptoms on the left side.

**Figure 1:**
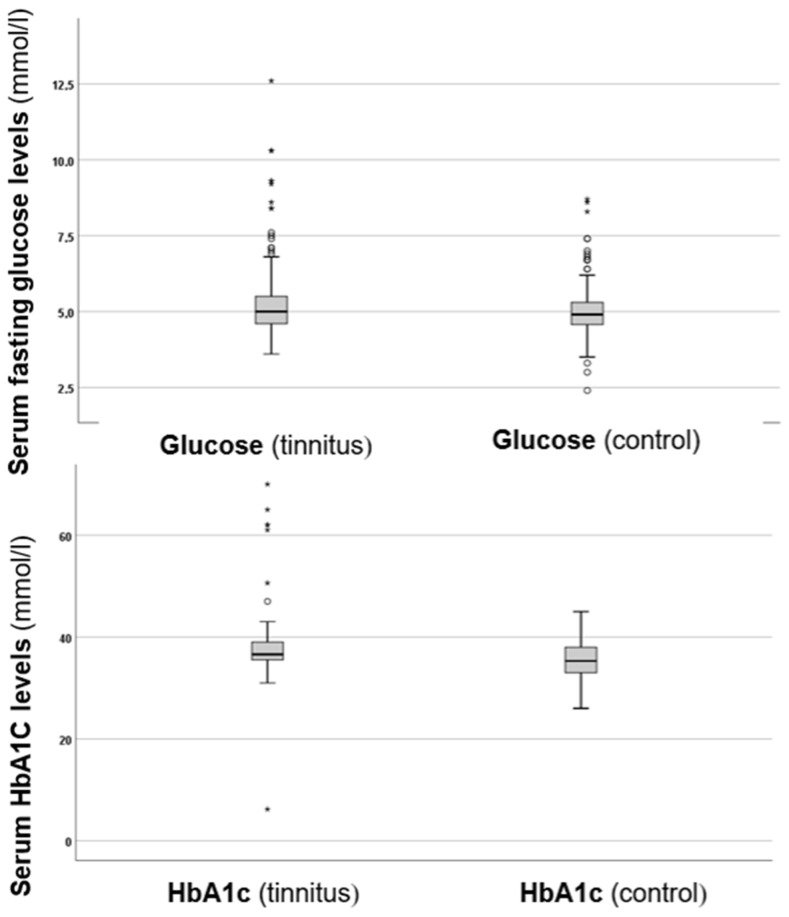
Boxplots presenting the serum fasting glucose and HbA1c levels in the control and tinnitus groups. In the boxplots, the boxes indicate the interquartile range of the data, while the whiskers show the lower and upper quartiles. The black line that separates the boxes marks the median values. The statistical differences were analysed using the Mann–Whitney *U* test (*p* < 0.05). HbA1c = haemoglobin A1C, mmol = millimole, L = litre. The asterisks and circles in the boxplot indicate the outliers.

In the next step of the investigation, serum fasting glucose and HbA1c levels were compared between the two groups, as shown in Figure 1.

As depicted in Figure 1, the tinnitus group exhibited higher fasting glucose levels compared to the control group. Analysis of the glucose levels revealed a statistically significant difference (*p* = 0.024 *), with higher values in the tinnitus group. Additionally, when examining HbA1c levels, the tinnitus group also showed higher values, which were statistically significant (*p* = 0.006 *). These results suggest that there are disturbances in glucose metabolism associated with tinnitus.

**Figure 2:**
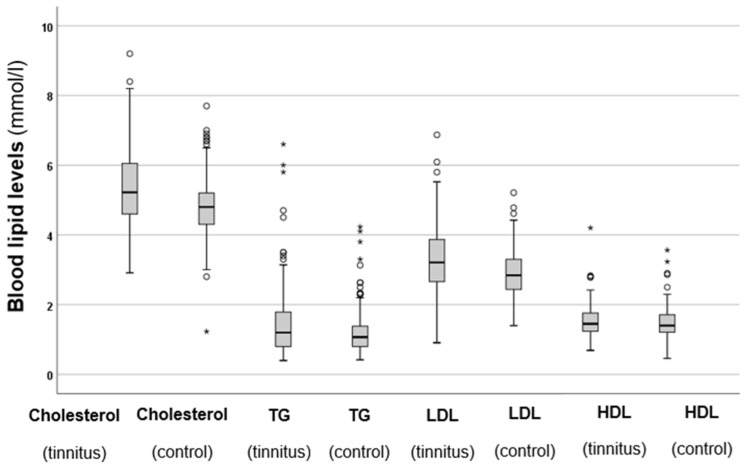
Total cholesterol, TG, LDL, and HDL levels in the tinnitus and control groups. In the boxplots, the boxes indicate the interquartile range of the data, while the whiskers show the lower and upper quartiles. The black line that separates the boxes marks the median values. The statistical differences were analysed using the Mann–Whitney *U* test (*p* < 0.05). HDL = high-density lipoprotein, LDL = low-density lipoprotein, mmol = millimole, L = litre, TG = triglycerides. The asterisks and circles in the boxplot indicate the outliers.

Serum cholesterol, TG, LDL, and HDL levels were also analysed, and the results are shown in Figure 2.

The results shown in Figure 2 indicate that the tinnitus group had significantly higher total cholesterol levels (*p* < 0.001 *) compared to the control group. Similarly, the tinnitus patients exhibited significantly elevated TG levels (*p* < 0.001 *) in comparison to those in the control group. A similar trend was observed for LDL levels, which were also significantly higher in the tinnitus group (*p* < 0.001 *). However, when it came to HDL levels, no statistically significant difference was found between the two groups (*p* = 0.984). These results indicate that patients with elevated cholesterol, TG, and LDL levels are at a higher risk of developing tinnitus. Additionally, the elevated levels of HDL, which provide protective benefits, are not observed in individuals with tinnitus.

**Figure 3:**
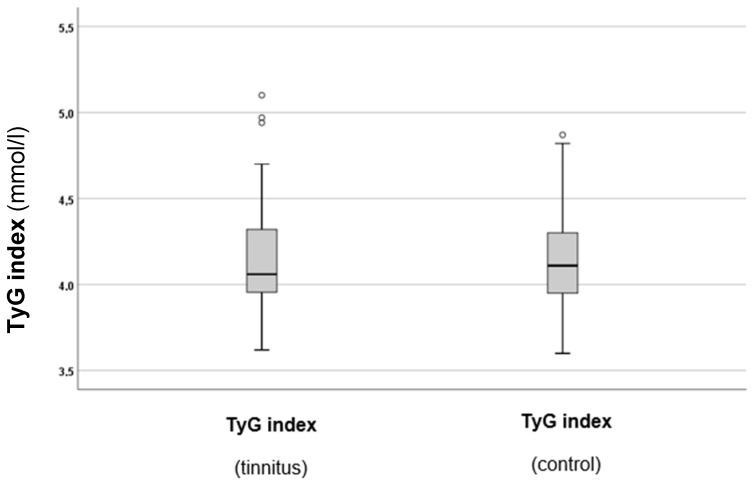
TyG indexes in the tinnitus and control groups. In the boxplots, the boxes indicate the interquartile range of the data, while the whiskers show the lower and upper quartiles. The black line that separates the boxes marks the median values. The statistical differences were analysed using the Mann–Whitney *U* test (*p* < 0.05). mmol = millimole, L = litre, TyG = triglyceride–glucose index. The circles in the boxplot indicate the outliers.

As a next step, the TyG index levels in both groups were calculated and compared. The results are summarised in Figure 3.

As can be observed from Figure 3, there was a slight tendency for higher TyG index values in the tinnitus group. However, statistical analysis did not indicate a significant difference between the two groups (*p* = 0.688). Therefore, additional analysis of the TyG index and other parameters was conducted subsequently.

**Figure 4:**
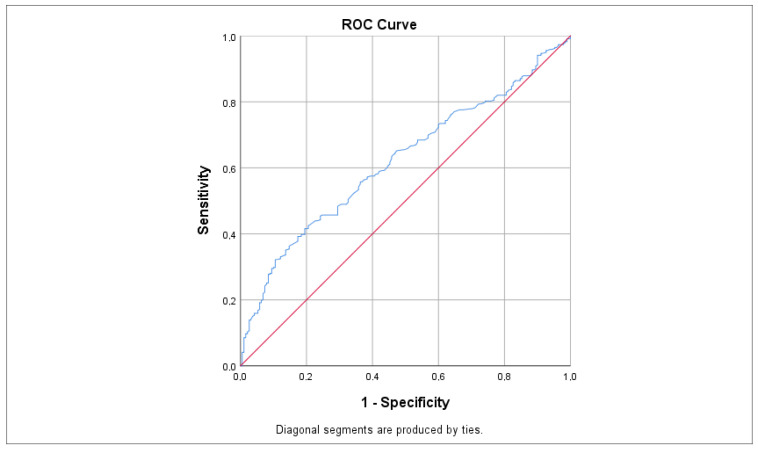
ROC curve demonstrating the sensitivity of LDL levels in predicting tinnitus. Sensitivity values were calculated using the AUC. The red line represents the reference line (random chance line) at 0.5, while the blue line indicates the ROC curve for LDL levels. AUC = area under the curve, ROC = receiver operating characteristic.

ROC analysis, as illustrated in Figure 4 and Table 2, indicated that LDL had the highest sensitivity value for predicting tinnitus at 0.620, which was statistically significant (*p* < 0.001 *; 95% CI: 0.573–0.668). Other significant predictors included total cholesterol (AUC = 0.603; *p* < 0.001 *, 95% CI: 0.557–0.648), HbA1c (AUC = 0.596; *p* = 0.006 *, 95% CI: 0.531–0.661), TG (AUC = 0.586; *p* < 0.001 *, 95% CI: 0.540–0.632), and glucose (AUC = 0.553; *p* = 0.023 *, 95% CI: 0.508–0.597), each with slightly lower sensitivity values. In contrast, the TyG index showed a non-statistically significant difference with a sensitivity value of 0.514 (*p* = 0.688; 95% CI: 0.443–0.585). Nonetheless, the sensitivity of the TyG index was fairly close to the sensitivity values of the other parameters.

To further investigate, the potential relationships between glucose levels and blood lipid parameters, along with basic factors related to tinnitus, such as the participants’ age, sex, symptom onset, and total THI scores were analysed. The results are presented in Table 3.

Table 3 summarises that serum glucose levels show a significant positive correlation with age (*p* < 0.001 *) and the onset of tinnitus (*p* = 0.034 *). These findings suggest that elevated serum glucose levels are associated with greater age and an earlier onset of tinnitus. In other words, older patients are more likely to exhibit increased glucose levels and experience tinnitus, and higher glucose levels may be a risk factor for prolonged tinnitus. Additionally, HbA1c values were significantly correlated (*p* < 0.001 *) with age, indicating that older patients tend to have higher levels. Considering blood lipids, both total cholesterol (*p* = 0.002 *) and TG (*p* = 0.020 *) were significantly correlated with age, indicating a risk for elevated blood lipid levels in older patients. Age was significantly correlated with tinnitus onset (*p* < 0.001 *), indicating that older patients experience more prolonged tinnitus, highlighting the relevance of more significant risk factors. Other parameters did not show a significant correlation with each other; thus, they were excluded from the table. Considering the effects of sex, statistically significant (*p* = 0.003 *, Mann–Whitney *U* test) differences were observed in HbA1c levels between men (mean ± SD, 37.2 ± 5.1 mmol/L) and women (mean ± SD, 39.1 ± 4.8 mmol/L), indicating significantly higher values in women. A similar pattern (*p* = 0.032 *, Mann–Whitney *U* test) was found for total cholesterol levels (5.36 ± 0.95 mmol/L vs. 5.11 ± 0.92 mmol/L), with women reporting significantly higher values. However, in the case of TG, men (1.68 ± 0.84 mmol/L vs. 1.41 ± 0.64 mmol/L) presented significantly higher values (*p* = 0.006 *, Mann–Whitney *U* test). Interestingly, total THI scores, which refer to tinnitus severity, were not correlated with either parameter; therefore, they were not included in the table.

To further analyse the potential factors affecting tinnitus severity, a multinomial logistic regression model was applied. The results are presented in Table 4.

Table 4 shows that the occurrence of tinnitus was significantly influenced by elevated HbA1c levels (*p* = 0.007 *; OR: 4.435, 95% CI = 1.501–13.101) and TG levels (*p* = 0.001 *; OR: 27.262, 95% CI = 11.658–63.749). Other parameters did not have a significant effect on tinnitus occurrence according to the model. Furthermore, the occurrence of chronic tinnitus was significantly influenced by elevated glucose levels (*p* = 0.026 *; OR: 0.169, 95% CI = 0.035–0.813). Consequently, when adjusting for age and sex, HbA1c and TG levels had the most significant impact on the occurrence of tinnitus and the development of chronic tinnitus.

## 4. Discussion

The findings of this investigation indicate that individuals with subjective tinnitus have significantly higher levels of serum fasting glucose, HbA1c, and blood lipids, including total cholesterol, TG, and LDL, compared to healthy controls. Although the TyG index also showed higher values in individuals with tinnitus, this difference was not statistically significant. In predicting tinnitus, LDL displayed the highest sensitivity according to the ROC analysis. These results highlight the relationship between tinnitus and cardiovascular risk.

Only a limited number of studies have explored the relationship between glucose metabolism and tinnitus. Furthermore, in most cases, the specific glucose and HbA1c levels were not analysed and compared with healthy controls. One particular study found a significant link between patients’ age, diabetes mellitus, and the severity of tinnitus. The findings indicated that diabetes mellitus was associated with a 26% increased likelihood of developing tinnitus. The authors attributed this correlation to potential neuropathy or microangiopathy affecting the inner ear [14]. Elevated glucose levels in the inner ear can lead to several effects, which can be attributed to various factors. These include histopathological changes in the basement membrane of the stria vascularis and the basilar membrane of the organ of Corti. Additionally, a loss of spiral ganglion cells has also been observed [15]. It has been suggested that insulin resistance in the inner ear may contribute to dysfunction in the inner ear related to diabetes mellitus [16]. Tinnitus is influenced not only by vascular risk factors affecting the inner ear but also by central factors. For example, a study revealed that patients with tinnitus showed reduced cerebral blood flow, particularly in auditory regions of the brain. This decrease in blood flow may be linked to elevated glucose levels [17]. Notably, in the current investigation, no correlation was found between the severity of tinnitus and these vascular risk factors. The correlations with participants’ age align with the current study findings, indicating a higher risk for tinnitus and vascular risk factors with ageing. Another investigation suggested that chronic tinnitus may lead to changes in cerebral glucose metabolism, potentially accelerating cognitive impairment [18]. Elevated glucose levels and diabetes mellitus are associated with both sensorineural hearing loss and noise-induced hearing loss [19]. However, it is important to note that not all cases of tinnitus are linked to sensorineural hearing loss. This correlation emphasises the significance of hearing testing for individuals experiencing tinnitus. Furthermore, these results refer to a complex relationship between tinnitus and glucose metabolism.

Several studies have been conducted to examine the relationship between blood lipid levels and tinnitus. One study found that 13% of patients with tinnitus also had hyperlipidaemia, which doubled their risk of experiencing tinnitus. However, it is important to note that specific blood lipid levels were not included in that investigation [20]. One investigation found that elevated blood lipid levels may not be directly linked to tinnitus [21]. Despite this, the study emphasised the importance of cardiovascular risk factors. Tinnitus could serve as a warning signal that should not be ignored. Another investigation found a 1.27 higher odds ratio for tinnitus in the group with hypertriglyceridaemia, and a 1.21 times higher odds ratio in the group with a higher total cholesterol/HDL ratio [22]. A recent study found that low serum HDL levels were linked to depressive symptoms in patients with chronic tinnitus [23]. A study using peripheral blood samples found that hypercholesterolaemia increases the risk of tinnitus. Additionally, there was an 8-fold higher risk associated with both smoking and hypercholesterolaemia, and a 3.5-fold higher risk when hypercholesterolaemia occurred alongside diabetes mellitus [24]. In a study examining treatment effectiveness, it was found that using 10 mg of atorvastatin daily led to improvements in tinnitus symptoms. Additionally, most participants experienced enhancements in their blood lipid profiles [25]. Alterations in blood lipid levels can affect the inner ear based on different mechanisms. One possible explanation for how dyslipidaemia may contribute to tinnitus development involves the blockage of capillaries in the rigid blood vessels within the inner ear. This blockage can lead to biochemical changes in the endolymph, resulting in ischaemia of the inner ear [26]. A decrease in blood flow to the stria vascularis can cause sensorineural hearing loss, which is often accompanied by tinnitus [27]. Additionally, elevated blood lipid levels can result in lipid accumulation in the cell membranes of outer hair cells, leading to chronic hypoxia and disrupted cellular metabolism [28].

Our current understanding indicates that tinnitus is not exclusively linked to the inner ear. MRI scans have revealed structural changes in the thalamus, auditory brainstem, and auditory cortex of patients with tinnitus. Additionally, damage to the cochlea results in increased spontaneous firing rates in various auditory structures, including the inferior colliculus and both the primary and secondary auditory cortex, although this does not necessarily affect the auditory nerve. In addition to neural hyperactivity, increased neural synchrony and bursting activity are essential mechanisms. It is important to note that even clinically undetectable and symptom-free cochlear damage can lead to tinnitus and its related pathologies. The downregulation of GABAergic inhibition has been suggested as a potential factor contributing to hyperactivity. However, it is important to note that several non-auditory regions, for instance, the limbic system, also play crucial roles in the development of tinnitus. The plasticity of limbic circuits is crucial in developing chronic persistent tinnitus [29].

Given the role of the central nervous system on tinnitus development, it is crucial to evaluate factors beyond blood lipid and glucose metabolism in the inner ear. Hypercholesterolaemia is known to increase cognitive impairment, provoke cerebrovascular dysfunction, and result in astrogliosis and microgliosis, leading to white matter inflammation and neuroinflammation. Changes in brain cholesterol metabolism play a role in the development of Alzheimer’s, Parkinson’s, and Huntington’s diseases. Additionally, cerebral total cholesterol, LDL, and TG levels influence neurotransmitters, leading to an increase in glutamate, dopamine, and N-methyl-D-aspartate (NMDA) levels while decreasing gamma-aminobutyric acid (GABA), serotonin (5-HT), and low-density lipoprotein receptor (LDLR) levels [30]. GABA is a well-established inhibitory neurotransmitter in the central auditory system. Therefore, a decrease in GABA levels contributes to neuroexcitatory effects. However, glutamate acts as an excitatory neurotransmitter in the auditory system, leading to increased neuronal excitability when its levels rise [31]. These associations underscore the importance of lipid metabolism in tinnitus not solely based on inner ear effects but based on neurotransmission in the central nervous system. Research has shown that glucose levels can significantly impact neurotransmission. In particular, high glucose levels can lead to increased glutamate levels, which is primarily an excitatory neurotransmitter [32]. Studies have indicated that patients with diabetic neuropathy exhibit significantly higher levels of glutamate and glutamine while GABA levels are notably lower [33]. This finding emphasises the role of metabolic disorders in neurotransmission. Additionally, given the strong relationship between tinnitus and vascular risk factors, this information may enhance our understanding of the pathophysiology of tinnitus and inform potential therapeutic approaches.

To our knowledge, there have been no studies specifically examining the relationship between tinnitus and the TyG. Furthermore, it should be noted that although a trend of higher TyG index values was observed in the tinnitus group, the difference compared to controls was not statistically significant. Therefore, the role of TyG index in predicting tinnitus remains uncertain. Only a limited number of investigations have looked into TyG in relation to hearing loss. For example, one study found a linear positive correlation between TyG levels and hearing loss, especially at higher frequencies [34]. This result is particularly significant because high-frequency tinnitus is often seen in everyday practice, frequently associated with high-frequency sensorineural hearing loss. Additionally, a Mendelian randomisation approach revealed an L-shaped association between the TyG index, glucose levels, and sensorineural hearing loss in the American population [35]. In this investigation, although tinnitus patients showed a tendency for higher TyG levels, no statistically significant difference was found. Therefore, further analysis of TyG levels in tinnitus is warranted.

The strengths of this investigation include the large number of tinnitus cases examined, the presence of a control group, and the analysis of specific glucose and blood lipid parameters alongside the TyG index. However, the study also has limitations. Firstly, the effects of treatment were not analysed, so there is no information regarding the improvement of tinnitus in relation to blood glucose and lipid disturbances. Additionally, the wide variation in the duration of tinnitus—ranging from a few weeks to several years—may introduce bias into the results. Furthermore, the cross-sectional design of the study also has limitations. Longitudinal studies would provide stronger evidence regarding whether metabolic disturbances could onset before tinnitus.

## 5. Conclusions

The findings of this study highlight the importance of changes in glucose and blood lipid metabolism in the development of tinnitus. Increased levels of blood glucose and lipids, especially LDL, are linked to tinnitus. Therefore, we recommend that clinicians screen for and address cardiovascular risk factors, including disturbances in glucose metabolism. Our findings also suggest the necessity of a comprehensive approach to managing tinnitus symptoms, rather than focusing solely on ear-related conditions. This perspective should be considered when developing therapeutic pathways for tinnitus. Given the cross-sectional design of the study, which may limit the results, further longitudinal studies are necessary.

## Figures and Tables

**Table 1:** Participants’ basic information. SD = standard deviation. ^†^ Mann–Whitney *U* test, ^††^ Chi- square test (*p* < 0.05).

Parameter	Tinnitus Group	Control Group	*p*-Value
Age (mean years ± SD)	51.81 ± 12.8	49.02 ± 8.77	0.06 ^†^
Sex (men/women)	179/235	116/158	0.81 ^††^
Tinnitus onset (mean months ± SD)	36.17 ± 2.88		
Tinnitus location			
Right, *n* (%)	103 (24.9%)		
Left, *n* (%)	131 (31.6%)		
Bilateral, *n* (%)	180 (43.5%)		

**Table 2:** Parameters of the ROC curve analysis. AUC = area under the curve, CI = confidence interval, HbA1c = haemoglobin A1C, LDL = low-density lipoprotein, ROC = receiver operating characteristic, Std. = standard, TG = triglycerides, TyG = triglyceride–glucose index. The asterisk (*) denotes a statistically significant difference.

	AUC	Std. Error	*p*-Value	95% CI (Lower Bound)	95% CI (Upper Bound)
**TyG**	0.514	0.036	0.688	0.443	0.585
Glucose	0.553	0.023	0.023 *	0.508	0.597
HbA1c	0.596	0.033	0.006 *	0.531	0.661
Total cholesterol	0.603	0.023	<0.001 *	0.557	0.648
TG	0.586	0.024	<0.001 *	0.540	0.632
LDL	0.620	0.024	<0.001 *	0.573	0.668

**Table 3:** Correlation between tinnitus-related parameters and glucose and blood lipid parameters using the Spearman’s correlation test (*p* < 0.05 *). HbA1c = haemoglobin A1C, TG = triglycerides. Please note that only the correlations deemed statistically significant are presented in the table.

Parameter	Spearman’s Rho	*p*-Value
Glucose–age	0.338	<0.001 *
Glucose–tinnitus onset	0.107	0.034 *
HbA1C–age	0.458	<0.001 *
Cholesterol–age	0.156	0.002 *
TG–age	0.121	0.020 *
Age–tinnitus onset	0.344	<0.001 *

**Table 4:** A multinomial logistic regression model adjusted for age and sex, using tinnitus occurrence and chronic tinnitus (defined as lasting more than 3 months) as dependent variables. CI = confidence interval, HbA1c = haemoglobin A1C, HDL = high-density lipoprotein, LDL = low-density lipoprotein, OR = odds ratio, Std. = standard, TG = triglycerides, TyG = triglyceride–glucose index. The significant results (*p* < 0.05) are indicated with an asterisk (*).

Dependent	Predictor	*β*	Std. Error	*p*-Value	OR	95% CI (Lower Bound)	95% CI (Upper Bound)
Tinnitus occurrence	HbA1c	1.489	0.531	0.007 *	4.435	1.501	13.101
	Glucose	−0.173	0.356	0.627	0.841	0.418	1.691
	Cholesterol	0.673	0.605	0.266	1.960	0.598	6.420
	TG	3.305	0.433	0.001 *	27.262	11.658	63.749
	LDL	0.435	0.642	0.498	1.544	0.439	5.435
	HDL	−0.086	0.251	0.732	0.918	0.561	1.500
	TyG	0.392	0.443	0.377	1.480	0.621	3.527
Chronic tinnitus (lasting over 3 months)	Glucose	−1.777	0.801	0.026 *	0.169	0.035	0.813

## Data Availability

The original contributions presented in this study are included in the article. Further inquiries can be directed to the corresponding author.

## Abbreviations

The following abbreviations are used in this manuscript:

AUC	Area under the curve
CI	Confidence interval
dL	Decilitre
GABA	Gamma-aminobutyric acid
HBA1c	Haemoglobin A1C
HDL	High-density lipoprotein
5-HT	Serotonin
IBM	International Business Machines Corporation
L	Litre
LDL	Low-density lipoprotein
LDL-R	Low-density lipoprotein receptor
Ln	Natural logarithm
mmol	Millimole
MRI	Magnetic resonance imaging
NMDA	N-methyl-D-aspartate
ROC	Receiver operating characteristic
SD	Standard deviation
SPSS	Statistical Package for the Social Sciences
TG	Triglyceride
TyG	Triglyceride–glucose index
THI	Tinnitus Handicap Inventory
